# Modeling and simulation of phototransduction cascade in vertebrate rod photoreceptors

**DOI:** 10.1186/s12886-019-1048-7

**Published:** 2019-02-20

**Authors:** Guofeng Pan, Jinglu Tan, Ya Guo

**Affiliations:** 1 Ministry of Education, Key Laboratory of Advanced Process Control for Light Industry, Jiangnan University, Wuxi, 214122 China; 2 University of Missouri, Columbia, MO 65211 USA

**Keywords:** Phototransduction, ERG a wave, Rod, Photoreceptor, Kinetic model structure

## Abstract

**Background:**

The activation of phototransduction cascade in rod photoreceptors has been well studied in literature, but there is a lack of a mature kinetic model structure covering both the activation and inactivation processes.

**Methods:**

In this work, a kinetic model structure is developed to describe the major activation and inactivation processes in vertebrate rod photoreceptors with the electroretinogram (ERG) as output. Simulation was performed to validate developed model structure.

**Results:**

The developed model structure could fit experimental data with small error.

**Conclusions:**

The result indicated that the developed model structure could show the inactivation process of phototransduction cascades in the rod photoreceptors.

## Background

When the molecules of rod photoreceptors, rhodopsin, absorb photons, a cascade of reactions are triggered [10, 13]. This eventually results in the decline of intracellular *cGMP* concentration and closure of *cGMP* gated Na^+^/Ca^2+^ ion channels in plasma membrane of rods, and leads to changes of electrical current and voltage [17]. The electrical current or voltage from the photoreceptors is an important part of electroretinogram (ERG), which is the summation of a few components of extracellular currents from different cells [13, 25, 28]. ERG has become an important diagnosis tool as the measurement standard protocols are developed and commercial instruments are available [8, 12, 19, 20, 22]. The response in the photoreceptor is very important to vision because it is the early stage of ERG and triggers latter vision activities [2, 5, 7, 16].

In literature, there are many efforts on modeling and simulation of phototransduction cascade in rods and ERG generation [6, 9, 11, 12, 16, 17, 21, 26, 27]. A recent review on modeling the retina in health, development and disease was well conducted by Roberts et al. [24]. Phenomenological analytical functions or stochastic events are often used to simulate the processes. These efforts have significantly contributed to the understanding of phototransduction cascade and vision system. However, there are limitations if they are applied for the estimation of reaction rates from measured ERG signal because the reaction rates are not explicit variables in phenomenological model structure and there are many difficulties (e.g. computation speed) in estimating model parameters from stochastic event models. The models in literature usually only focus on the activation processes. Estimation of reaction rates from measured ERG signal can be very useful for diagnosis; therefore, it is meaningful to develop a kinetic model structure with reaction rates as parameters for both the activation and inactivation processes. A kinetic model structure for this purpose should be fundamentally based on major reaction kinetics but not include too many reaction details; otherwise, the complexity from the details will incur difficulty in parameter estimation algorithm convergence and reduce computation speed. Currently, there is a lack of a mature kinetic model structure covering both the activation and inactivation processes. In this work, a kinetic model structure is developed, which included the major reactions phototransduction cascade. The capability of the model structure to represent the inactivation process has been demonstrated.

## Method

Based on published work on the molecular mechanisms for phototransduction cascade in vertebrate rod photoreceptors, the involved major activation and inactivation biochemical reactions are summarized and the model structure is developed.

### Activation of rhodopsin

A captured photon may set an inactivated rhodopsin (*R*) to its activated state (R*) by isomerizing its chromophore from 11-*cis* to the all-*trans* form [1, 12, 13]. The production rate of R* is proportional to the concentration of *R* and light intensity *u*, which can be represented by:

1 $$ R\kern0.5em \overset{k_1u}{\to}\kern0.5em {R}^{\ast } $$

where *k*_1_ is the activation rate of rhodopsin.

### Activation of G-protein (G-GDP) by activated rhodopsin

Through diffusion on the disc membrane, R* and inactivated G-GDP interact, which results in a series of reactions including [12, 16]: (*1*) R* binds to G-GDP and R*-G-GDP is formed, (*2*) GDP is released and R*-G is formed, (*3*) GTP binds to R*-G and R*-G-GTP is formed, (*4*) R* is released and G-GTP is formed, which then separates into submits G_*βγ*_ and G_α_*-GTP. G_α_*-GTP is activated and can trigger further reactions. R* is not altered in the processes and serves as an enzyme. For simplification, *G* is used to denote G-GDP and G* is used to denote the activated G_α_*-GTP. It is not practical to include all the reaction details; otherwise, the complexity of the model structure will be dramatically increased. According to Michaelis-Menten kinetics, the intermediate reactions are simplified and these reactions can be approximately represented as:

2 $$ {R}^{\ast }+G\underset{k_3}{\overset{k_2}{\rightleftarrows }}{C}_1\overset{k_4}{\to }{R}^{\ast }+{G}^{\ast } $$

where *C*_1_ is the intermediate complex formed by the binding of R* and *G*, and *k*_2_, *k*_3_, and *k*_4_ are the reaction rate constants.

### Activation of phosphodiesterase (PDE) by activated G*

Two G* units bind to the two inhibitory subunits of inactivated PDE (denoted as *E*) and activate the *α* and *β* catalytic subunits of PDE and make PDE in active state [1, 12]; however, the two PDE subunits seem to work independently. If E* is used to denote a single activated subunit of PDE, the activation process of PDE can thus be represented as [11]:

3 $$ {G}^{\ast }+E\kern0.5em \overset{k_5}{\to}\kern0.5em {E}^{\ast } $$

where *k*_5_ is the activation rate of *E*.

### Change of cyclic GMP (cGMP) concentration

E* catalyzes the hydrolysis of *cGMP* (denoted as *cG* for conciseness) and produces *GMP* [23]. According to Michaelis-Menten kinetics, this process can be represented as:

4 $$ {E}^{\ast }+ cG\underset{k_7}{\overset{k_6}{\rightleftarrows }}{C}_2\overset{k_8}{\to }{E}^{\ast }+ GMP $$

where *C*_2_ is the intermediate complex formed by the binding of E* and *cG*, and *k*_6_, *k*_7_, and *k*_8_ are the reaction rate constants.

### Inactivation of E* and G*

The GTP bound to G* in the complex of E* is hydrolyzed to GDP, which results in separation of the complex, production of PDE and G_α_-GDP (*G*_*r*_), and inactivation of both E* and G*. *G*_*r*_ will finally convert to G-GDP (*G*) [1, 6]. These processes can be represented as:

5 $$ {E}^{\ast}\overset{k_9}{\to }{G}_r+E $$

6 $$ {G}_r\overset{k_{10}}{\to }G $$

where *k*_9_ is the rate of E* hydroxylation and *k*_10_ is the rate of *G*_*r*_ converts to *G*.

### Channel activity, R* inactivation, and cGMP restoration

The decrease of *cGMP* concentration as represented in Eq. (4) leads to closure of *cGMP* gated channels and reduction of Ca^2+^ influx, and thus cytoplasmic Ca^2+^ concentration is dropped. If *Ca* is used to denote the cytoplasmic Ca^2+^ concentration, the drop of Ca^2+^ concentration can be represented as:

7 $$ \frac{d(Ca)}{dt}=-{k}_{11}\left({cG}_0^{ncG}-{cG}^{ncG}\right) $$

where *cG*_0_ is the concentration of *cGMP* after dark-adaption, *cG* is the current *cGMP* concentration, *k*_11_ is a constant, *n*_cG_ is the Hill coefficient that describes *cGMP* opening channel in a cooperative manner [1]. In this work, *n*_cG_ is set as 2 [12].

The reduced Ca^2+^ concentration causes recoverin (a guannylyl cyclase activation protein, GCAP) to release its Ca^2+^ and separate from rhodopsinkinase (RK). The increase of Ca^2+^ concentration is initiated by Ca^2+^ concentration drop from the dark-adapted value *Ca*_0_ and is assumed as proportional to the concentration drop, which implies that the concentration change can be represented as:

8 $$ \frac{d(Ca)}{dt}={k}_{12}\left({Ca}_0- Ca\right) $$

where *k*_12_ is the rate that *Ca* is increased.

The free form of RK phosphorylizes R* and allows arrestin (Arr) to bind and inactivate R*. The inactivation of R* in this way is mediated by Ca^2+^ and originates from the reduction of Ca^2+^ concentration. For simplicity, the inactivation of R* is assumed as proportional to the Ca^2+^ concentration drop from its dark-adapted value Ca_0_ and thus can be represented as:

9 $$ {R}^{\ast}\overset{k_{13}\left({Ca}_0- Ca\right)}{\to }R $$

where *k*_13_ is the deviation rate of R* mediated by the reduction of Ca^2+^ concentration.

The Ca^2+^-free form of GCAP will bind to gyanylyl cyclase (GC) and turn on the enzymatic activity of GC, which catalyzes the synthesis of *cGMP* from GTP [16, 23]. According to Biernbaum and Bownds [3], GTP concentration change only occurs when light intensity is stronger than the saturation level and much later than the GC catalyzed *cGMP* production. In this work, the concentration of GTP is thus considered as a constant. Again, this activity is initiated and mediated by Ca^2+^. For simplicity, the synthesis of *cGMP* is assumed as proportional to the Ca^2+^ concentration drop from its dark-adapted value *Ca*_0_ and the drop of *cG* concentration from its dark-adapted value *cG*_0_. It is thus the change of *cG* concentration can be represented as:

10 $$ \frac{d(cG)}{dt}={k}_{14}\left({Ca}_0- Ca\right)\left({cG}_0- cG\right) $$

where *k*_14_ is the synthesis rate of *cGMP* mediated by the reduction of Ca^2+^ concentration.

Reduction of cGMP concentration will also cause cGMP buffer in the cytoplasm releasing free form of cGMP and the diffusion of cGMP under concentration gradient [23]. If these activities are assumed to proportional to the drop of cGMP concentration, the increase of free cGMP concentration in cytoplasm can be represented as:

11 $$ \frac{d(cG)}{dt}={k}_{15}\left({cG}_0- cG\right) $$

where *k*_15_ is a constant.

Independent of RK and Arr, R* may be inactivated in an abnormal and prolonged manner [12]. This process is represented as:

12 $$ {R}^{\ast}\overset{k_{16}}{\to }R $$

where *k*_16_ is the rate of R* deactivation that is not caused by the activities mediated by Ca^2+^ concentration change as in Eq. (9).

**Fig. 1:**
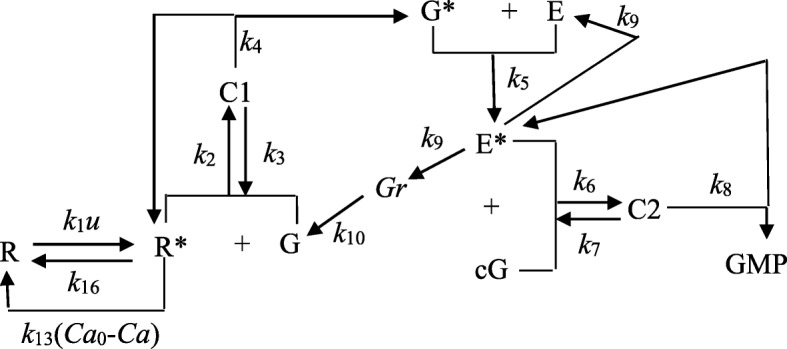
Major chemical reaction transition diagram (R-rhodopsin, R*-activated rhodopsin, G--G protein, G*-- activated G protein, E--phosphodiesterase or PDE, E*-- a single activated subunit of PDE, cG--cGMP, C1 and C2--intermediate complexes. Mass diffusion processes. *u* denotes light intensity. All the “*k*”s are reaction rates. Typically, they have the unit 1/s (first reaction kinetics) or mol/s (second reaction kinetics) depending on the unit used for the concentrations)

All major chemical reaction transitions are summarized in Fig. 1.

Let *x*_l_ through *x*_8_ denote the concentrations of R*, G*, *C*_1_, E*, *C*_2_, *cG*, *Gr*, and *Ca*^*2+*^ respectively. The concentration at the balance after dark-adaptation for *R*, *G*, *E*, *cG*, *Ca* are denoted as *R*_0_, *G*_0_, *E*_0_, *cG*_0_, *Ca*_0_, respectively. According to chemical reaction kinetics, reaction speed is usually proportional to species concentration or the probability that reactants meet each other, species concentration changes rate with time represented by the phototransduction cascade in Eqns. (1) through (12) can be written as:

13 $$ \frac{dx_1}{dt}={k}_{1u}\left({R}_0-{x}_1-{x}_3\right)-{k}_2{x}_1\left({G}_0-{x}_3-{x}_2-{x}_4-{x}_5-{x}_7\right)+{k}_3{x}_3+{k}_4{x}_3-{k}_{13}\left({Ca}_0-{x}_8\right){x}_1-{k}_{16}{x}_1 $$

14 $$ \frac{dx_2}{dt}={k}_4{x}_3-{k}_5{x}_2\left({E}_0-{x}_4-{x}_5\right) $$

15 $$ \frac{dx_3}{dt}={k}_2{x}_1\left({G}_0-{x}_3-{x}_2-{x}_4-{x}_5-{x}_7\right)-{k}_3{x}_3-{k}_4{x}_3 $$

16 $$ \frac{dx_4}{dt}={k}_5{x}_2\left({E}_0-{x}_4-{x}_5\right)-{k}_6{x}_4{x}_6+{k}_7{x}_5+{k}_8{x}_5-{k}_9{x}_4 $$

17 $$ \frac{dx_5}{dt}={k}_6{x}_4{x}_6-{k}_7{x}_5-{k}_8{x}_5 $$

18 $$ \frac{dx_6}{dt}=-{k}_6{x}_4{x}_6+{k}_7{x}_5+{k}_{14}\left({Ca}_0-{x}_8\right)\left({cG}_0-{x}_6\right)+{k}_{15}\left({cG}_0-{x}_6\right) $$

19 $$ \frac{dx_7}{dt}={k}_9{x}_4-{k}_{10}{x}_7 $$

20 $$ \frac{dx_8}{dt}=-{k}_{11}\left({cG}_0^{ncG}-{x}_6^{ncG}\right)+{k}_{12}\left({Ca}_0-{x}_8\right) $$

Changes of cGMP concentration (*x*_6_) lead to closing or opening of cGMP-gated ion channels and changes in the circulating current between the outer and inner segments of photoreceptor (Lam and Pugh, 2004; [16]). The deviation of current from the dark-value is proportional to the changes of closing and opening of cGMP-gated channels. The recorded electrical voltage can be represented as:

21 $$ f(t)={k}_{17}\left({cG}_0^{ncG}-{x}_6^{ncG}\right) $$

where *f*(*t*) is the measured ERG signal caused by light stimulation, *k*_17_ is a gain factor accounting for the number of photoreceptors and instrumentation gain (Because the produced current is proportional to the number of photoreceptors and instrumentation gain, the two factors will appear as product, which is merged into one term and cannot be separated.).

## Results

ERG data were reproduced from the publication of Lu et al. [16] and Lu [15]. ERG were recorded from three wild-type mice (61-day-oldm males), three NOB1 mice (healthy, 61-day-old), and three NOB1 mice with drug treatment (N-methy-N-nitrosourea). The bipolar cells of NOB1 mice were genetically disabled to suppress ERG b-wave. Details about the experiments can be found in Lu et al. [16]. The Levenberg-Marquardt method was used to estimate the model parameters (*k*_1_-*k*_17_) in this work, which was adopted from the Matlab codes in the appendix of Lu [15]. In the Levenberg-Marquardt method, the increment of each model parameter was computed according to the Jacobian matrix of partial derivatives of *f*(*t*) with respect to (*k*_1_-*k*_17_) evaluated at all data points, the error between the model prediction and experimental data, and a damping factor for improving algorithm convergence. The algorithm of the Levenberg-Marquardt method can be found in Marquardt [18], Levenberg [14], Constantinides and Mostoufi [4]. The Runge-Kutta algorithm was used to integrate the differentiation equations. Both the Runge-Kutta and the Levenberg-Marquardt were programmed in Matlab. The model parameters and the total or dark concentrations in Eqns. (13) through (21) can always be normalized by redefining the state variables and maintaining a constant *R*_0_. It is thus the ratios of the state variables define the shape of ERG. The ratios for the initial values for R_0_, G_0_, E_0_, cG_0_, and Ca_0_ were set as 50, 5, 0.5, 4, and 0.22, respectively. The initial values for the state variables of *x*_1_ through *x*_8_ were set as [0 0 0 0 0 cG_0_ 0 Ca_0_] since fully dark adaptation was applied before measurements. In this work, the values of total or dark concentrations (*G*_0_, *E*_0_, *cG*_0_, and *Ca*_0_) were also optimized in the algorithm while *R*_0_ was maintained as a constant for all the groups. By doing this, the number of unknown parameters was reduced by 1, which was significant to improving computation speed and algorithm convergence. Accordingly, the estimated model parameters were effective rates. The constant *R*_0_ value served as a reference and made the estimated effective rates comparable and could be used for classification as demonstrated in this work.

Although NOB1mouse and a chemical technique [16] were used to reduce ERG b-wave, part of the b-wave and/or other ERG components from cells other than rod photoreceptors still existed. Because the response of photoreceptors is the early part of ERG, only the initial data segment from the beginning of light stimulus to a moment slightly after ERG trough was used to estimate model parameters. In this way, the effect of ERG components from other cells was reduced since the proposed model structure was only for the phototransduction cascade in rod photoreceptors.

**Fig. 2:**
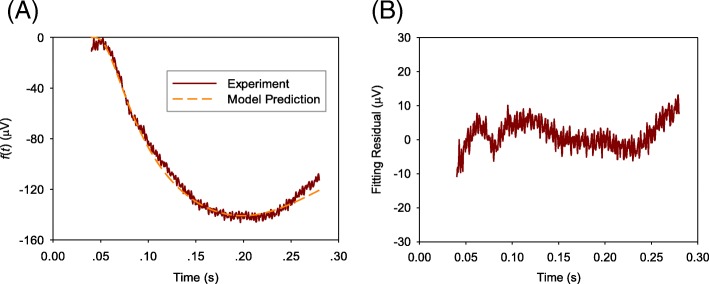
A comparison of averaged value of measured ERG data *f*(*t*) from healthy NOB1 mice with model prediction **a** and the fitting residual **b**

**Fig. 3:**
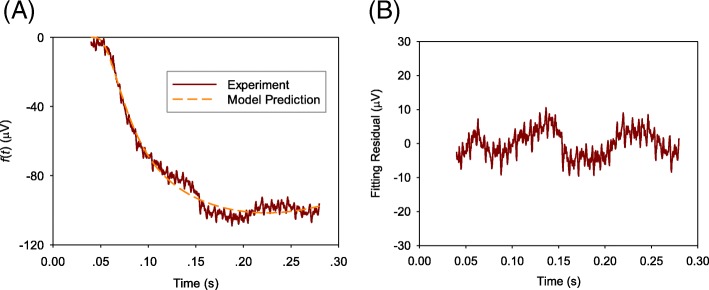
A comparison of averaged value of measured ERG data *f*(*t*) from photoreceptor-damaged NOB1 mice with model prediction **a** and the fitting residual **b**

**Fig. 4:**
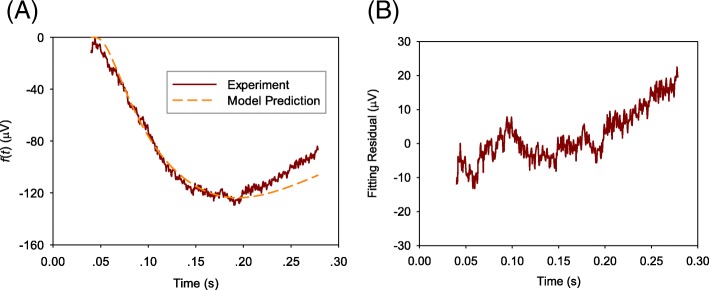
A comparison of averaged value of measured ERG data *f*(*t*) from wild-type mice with model prediction **a** and the fitting residual **b**

Figures 2, 3, and 4 show the comparisons of the averaged value of measured ERG data with model predictions and fitting residual for healthy NOB1, photoreceptor-damaged, and wild-type mice till 0.28 s, respectively. As shown in these figures, the model prediction could largely fit the initial stage of ERG, which is mainly from the rod photoreceptors. The relative fitting error for each of the three individual groups is less than 10% and the averaged value of relative errors for all the three groups of mice is 6.8%.

The mean value of *k*_1*u*_ to *k*_17_ for the three groups of mice are shown in Table 1.

**Table 1:** Means of *k*_1*u*_ to *k*_17_ for the three groups of mice

Model Parameter (or feature)	Groups	Values
*k*_1 *u*_	NOB	0.6717
	NOB with Drug	0.5488
	Wild	0.7487
*k*_2_	NOB	17.9455
	NOB with Drug	27.5449
	Wild	17.5549
*k*_3_	NOB	18.2341
	NOB with Drug	14.4897
	Wild	17.4280
*k*_4_	NOB	5847.3409
	NOB with Drug	3997.7881
	Wild	5723.8125
*k*_5_	NOB	52.3225
	NOB with Drug	40.9749
	Wild	51.0795
*k*_6_	NOB	0.2329
	NOB with Drug	2.3551
	Wild	0.2336
*k*_7_	NOB	45.6849
	NOB with Drug	34.2112
	Wild	45.7306
*k*_8_	NOB	36.8285
	NOB with Drug	24.9522
	Wild	36.2993
*k*_9_	NOB	6.1385
	NOB with Drug	5.4685
	Wild	6.0209
*k*_10_	NOB	1.2880
	NOB with Drug	1.2817
	Wild	1.2676
*k*_11_	NOB	52.8416
	NOB with Drug	35.9586
	Wild	53.0859
*k*_12_	NOB	31.2042
	NOB with Drug	27.3478
	Wild	28.8673
*k*_13_	NOB	79.5587
	NOB with Drug	56.7860
	Wild	77.7604
*k*_14_	NOB	14.9012
	NOB with Drug	2.1693
	Wild	14.7936
*k*_15_	NOB	11.0894
	NOB with Drug	9.0645
	Wild	10.6546
*k*_16_	NOB	6.5656
	NOB with Drug	3.1117
	Wild	5.5993
*k*_17_	NOB	1649.2012
	NOB with Drug	614.8300
	Wild	2227.1000

**Fig. 5:**
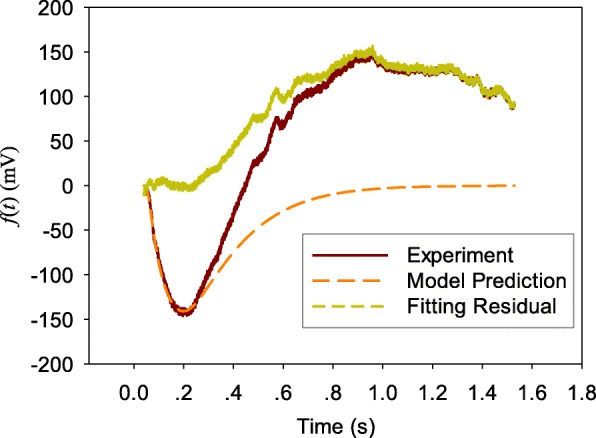
Simulation of rod photoreceptor response *f*(*t*) of a healthy NOB1 mouse for a longer time

**Fig. 6:**
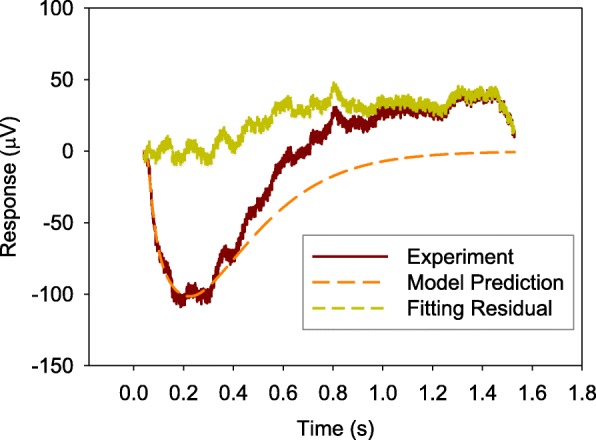
Simulation of rod photoreceptor response *f*(*t*) of a photoreceptor-damaged NOB1 mouse for a longer time

**Fig. 7:**
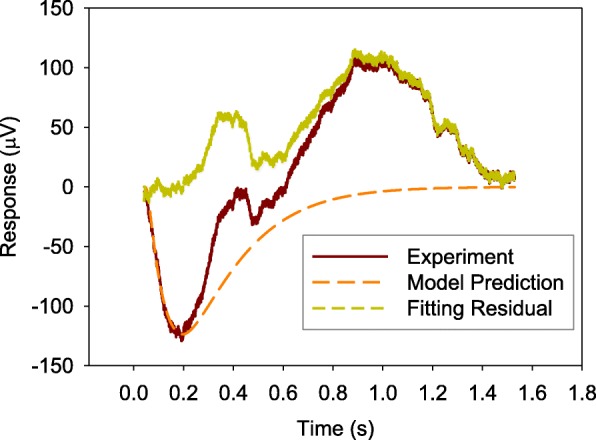
Simulation of rod photoreceptor response *f*(*t*) of a wild-type mouse for a longer time

In order to verify the capability of the model structure to represent the inactivation process of rod phototransduction cascade, the parameters estimated from the initial part of the ERG data were used to simulate the photoreceptor response for a much longer time. Figures 5, 6, and 7 show the simulation results, measured ERG data, and the difference between the simulated results and ERG data (fitting residual) for the three types of mice (healthy NOB1, photoreceptor-damaged NOB1, and wild-type), respectively. The simulated response from photoreceptors can match the initial part of ERGs and return the dark-adapted value after the stimulation light is turned off for a while, which agree with the long term behavior of rods and ERGs without the responses from other cells like bipolar cell [21]. The fitting residuals in the three figures can serve as predictions of the ERG components from cells other than rod photoreceptors.

## Discussions

Observations of Figs. 2, 3 and 4 reveal that the fitting errors mainly come from two sources. The first one is the high frequency noise on top of the signal, the other one is the discrepancy between experimental data and model prediction after the lowest trough of ERG. Because the existence of b-wave (generate later than the photoreceptor a-wave) at some degree makes ERG increase faster and the model structure does not cover the generation of ERG b-wave, the fitting error is larger at the later stage (after 0.2 s). The experimental data are bigger than model prediction for healthy NOB1 and wild-type subjects after the lowest trough of the ERGs. The fitting residual for wild-type group is stronger than the healthy NOB1 group, which suggests that the b-wave in ERG of the wild type group has more weight.

It is worth mentioning that the estimated reaction rates are effective rates because the initial concentrations were normalized. They are not comparable to the commonly reported pseudo first-order rates, but this does not affect the capability of the model structure to fit experimental data. In future research, further experiments including more subjects under other experimental conditions are needed to validate the developed model structure and compared model parameter changes with retina diseases.

## Conclusions

In this work, a kinetic model structure is developed to describe the major reactions of activation and inactivation of phototrasduction cascade in rods. Measured ERGs from three groups of mice (NOB1, photo-receptor damaged NOB1, and Wild-type) are used to validate the model structure. The developed model structure could fit experimental data with small error. The developed model structure can represent both the activation and inactivation activities in rod phototransductions. In future research, more effort is needed to validate and establish the developed model structure.

## Abbreviations

cGMP: cyclic guanosine monophosphate
ERG: Electroretinogram
G-GDP: G-protein
PDE: Phosphodiesterase
R: Rhodopsin
R*: activated rhodopsin
